# Preparation of (Nd, Ce)-Fe-B Regenerated Magnets by In-Situ Restoration of Grain Boundary Structure Using Nascent Nd-Fe-B Powder

**DOI:** 10.3390/ma17143381

**Published:** 2024-07-09

**Authors:** Xi Yu, Sangen Luo, Munan Yang, Qingpeng Shen, Honglong Yang, Shuwei Zhong, Weilong Zhang, Bin Yang

**Affiliations:** 1 College of Rare Earths, Jiangxi University of Science and Technology, Ganzhou 341000, China; gzyuxi@126.com (X.Y.); sangenluo@outlook.com (S.L.); shenqpshell@163.com (Q.S.); gtwy-yhl@139.com (H.Y.); zhongshuweidc@163.com (S.Z.); 2 Ganzhou Fortune Electronics Co., Ltd., Ganzhou 341000, China; 3 National Rare Earth Functional Material Innovation Center & Guorui Scientific Innovation Rare Earth Functional Materials Co., Ltd., Ganzhou 341000, China; 4 Key Laboratory of Development and Application of Ionic Rare Earth Resources, Ministry of Education, Ganzhou 341000, China; 5 Zhong Ke San Huan (Gan Zhou) New Material Co., Ltd., Ganzhou 341000, China; wilsonzhang@sanhuan-gz.com

**Keywords:** rare earth resource recycling, regenerated magnets, microstructure, sintering processes

## Abstract

Rare earth resource recycling is an important endeavor for environmental protection and resource utilization. This study explores the method of preparing regenerated magnets using waste magnets as raw materials based on existing processes. By utilizing existing Nd-Fe-B production equipment, various waste magnets are transformed into recycled powder. Next, nascent Nd-Fe-B powders with slightly higher rare earth content are selected as the repairing agent. The regenerated magnets are prepared by incorporating the nascent powder into the recycled powder. The focus lies in investigating the repairing effect of the nascent powder repairing agent on the microstructure of regenerated magnets and exploring the influence of sintering temperature and powder addition on the magnetic properties and microstructure of the regenerated magnets. The results showed that the nascent powder increased the proportion of grain boundary phases and effectively repaired the grain boundary structure of the regenerated magnets. In addition, the Pr element in the nascent powder replaces the Ce element in the recycled powder, which ultimately improves the magnetic properties of the regenerated magnet in a comprehensive manner. This study provides valuable insights and guidance for rare earth resource recycling and the preparation of regenerated magnets.

## 1. Introduction

Due to their excellent properties, such as high residual magnetization, high coercivity, and high magnetic energy products, sintered Nd-Fe-B permanent magnetic materials are widely used in high-tech, national defense, civil, household appliances, and industrial sectors [1,2,3,4]. Owing to the increasing demand for Nd-Fe-B magnets in the clean energy sector and other advanced technologies, the Nd-Fe-B magnet industry has experienced a global compound annual growth rate of 6.9% since 2010 [5]. From 2022 to 2035, the transition to clean energy, primarily driven by electric vehicles and wind power, is expected to be a key driver for the growth in demand for Nd-Fe-B magnets, with a projected global compound annual growth rate of 8.6% [6]. While rare earth elements are not expected to face shortages in the short term, numerous factors (limited rare earth ores, difficulty to mine, expensive heavy rare earths, difficult to recover resources, etc.) indicate the long-term sustainable supply of rare earths is challenging [7]. On the other hand, the substantial annual production accumulates a considerable quantity of existing Nd-Fe-B magnets, leading to a significant volume of scrap materials generated each year, which permeates the entire production line, including the crushing of bulk materials, pressing of powder, sintering of green compacts, and subsequent machining and usage processes [8]. Recovering rare earth elements through the recycling of waste Nd-Fe-B materials could be an important approach to alleviate the pressure on rare earth supply [9]. Therefore, the research and production of Nd-Fe-B waste recycling hold significant practical significance and broad prospects.

In recent years, significant efforts have been made to recover rare earth materials from waste Nd-Fe-B magnets. Currently, pyrometallurgy [10,11,12] is commonly employed to recover rare earth elements in the form of alloys or mixed compounds through high-temperature processes. However, this approach necessitates subsequent purification of the materials, and regrettably, pyrometallurgy is energy-intensive and unsuitable for treating oxidized waste. Another commonly used method is the hydrometallurgical treatment [13,14,15] of waste Nd-Fe-B magnets. However, during the leaching solution purification process, a large amount of chemicals is consumed, and a significant volume of waste liquids, such as oxalic acid and hydrochloric acid, are produced, which poses a severe burden on the environment. Lee et al. [16] proposed a method to tackle the recycling challenge associated with Nd-Fe-B magnets by demagnetizing and reusing them under air or argon atmospheres. The process involves initially heating the magnets to achieve demagnetization, followed by magnetization of the demagnetized material. The study’s results reveal that an increase in the demagnetizing temperature leads to a decrease in magnetic susceptibility, with a more pronounced decline observed in an air environment. Nevertheless, this approach necessitates that the waste magnets remain intact and readily processable, thereby presenting notable limitations. Another attempt at direct magnet recovery involves remelting the waste magnets and casting them into a master alloy, which is then used to produce new magnets [17]. For instance, Zakotnik et al. [18] collected old magnets and processed them into powder through hydrogen decrepitation (HD) and jet milling (JM) processes, followed by four repetitions of sintering. The study found that the density and magnetic properties gradually decreased with each repetition due to the gradual oxidation of the Nd-rich material and partial evaporation of Nd. However, the addition of Nd powder during the first repetition process demonstrated that the appropriate amount of Nd powder can maintain the density and magnetic properties of the recovered magnets. Zakotnik et al. [19] also pointed out that the maximum energy product (*BH*)_max_ obtained from recycled magnets is 15% lower than that of fresh magnets. Li et al. [20] processed waste magnets into powder through hydrogen decrepitation and ball milling and then incorporated NdH_x_ powder to manufacture regenerated magnets. The research results indicate that the addition of NdH_x_ powder can increase the proportion of the Nd-rich phase, and the magnetic properties are optimal when the doping amount is 2%. Compared to the original magnets, the coercivity recovery rate is 97.5%, the remanence recovery rate is 95.9%, and the maximum energy product recovery rate is 89.7%. Similarly, in a study by Liu et al. [21], the impact of nano-DyH_3_ on the microstructure and magnetic properties of recycled magnets was investigated. It was found that as the number of nano-DyH_3_ particles increased, the coercivity of the recycled magnets gradually increased. In comparison to the performance of the initial sintered waste magnets, the optimal recycled magnets contained 1.0 wt.% of nano-DyH_3_ particles. The recovery rates for coercivity, remanence, and (*BH*)_max_ were 101.7%, 95.4%, and 88.58%, respectively.

The waste Nd-Fe-B material is crushed into fine particles or powder and used in the powder metallurgy process to produce sintered Nd-Fe-B materials. This method, known as crushing and sintering [22,23], eliminates the need for mining and refining steps, reducing the production cycle. However, the magnetic properties of the magnets may decrease when the amount of waste exceeds 40% [24]. Despite the potential to produce high-performance recycled sintered Nd-Fe-B magnets through the use of hydrogen crushing technology for processing waste magnets and the subsequent incorporation of powder such as RE-rich alloys, DyF_3_, and NdHx to enhance grain boundary properties [21,25,26], the high cost and complexity present challenges for widespread large-scale application. Therefore, under the existing technological conditions of Nd-Fe-B manufacturers, this study aims to achieve the recycling of waste magnets without disrupting production lines. The high rare-earth-content fresh Nd-Fe-B powder, obtained by hydrogen decrepitation and airflow milling, is utilized to replace the traditional method of adding rich rare-earth alloys, nano DyH_x_, and NdH_x_ powder. The repair mechanism of grain boundaries in virgin powder has been investigated, leading to the successful development of high-performance recycled NdCe-Fe-B magnets. Furthermore, the effects of virgin powder addition and the sintering process on the magnetic properties and microstructure of sintered Nd-Fe-B recycled magnets have been systematically studied. Through these investigations, our aim is to provide theoretical support for the exploration of new pathways for the inexpensive, efficient, high-value, and pollution-free utilization of waste Nd-Fe-B magnets.

## 2. Materials and Methods

### 2.1. Experimental Procedure

**Figure 1:**
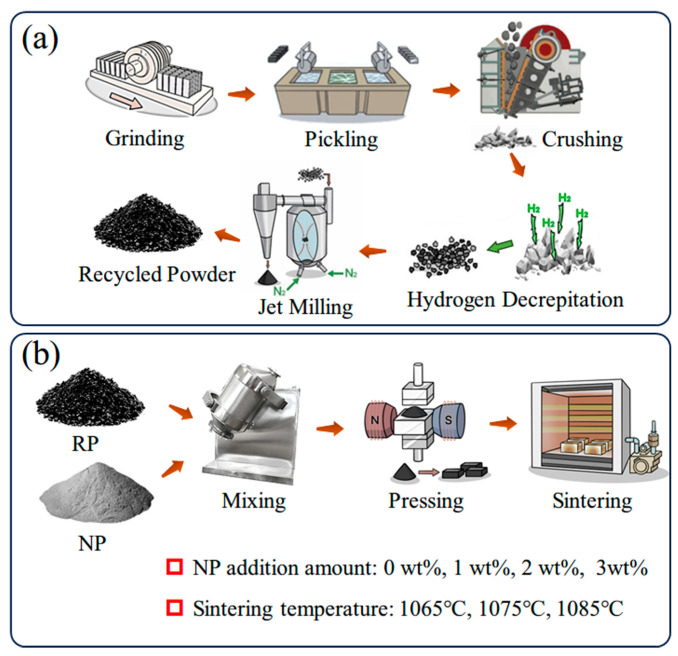
Experimental process: (**a**) Preparation process of NP powder; (**b**) preparation process of extruded pellets.

The waste magnets are collected from Ganzhou Fortune Electronic Co., Ltd. (Ganzhou, China), including machining scraps, long-term stored untreated and rusted blanks, as well as dismantled magnets from motors. These used magnets were prepared into powder and named recycled Nd-Fe-B powder (RP); the process is shown in Figure 1a. Firstly, the waste magnets are subjected to simple screening and then put into a vibration rust removal machine to eliminate the surface rust. Subsequently, the magnets were acid-washed in an aqueous solution of 3% HNO_3_ and 5% H_2_SO_4_ to further remove surface oxides and oil stains. The treated magnets are then roughly crushed in a crusher, and the pieces are quickly transferred to a hydrogen-crushing furnace for hydrogen decrepitation (HD). The procedure was to first heat the furnace to 180 °C for 20 min, then stop the heating and add hydrogen to the material to start the hydrogen absorption reaction. After 3 h, the hydrogen absorption was stopped, and dehydrogenation was started at a dehydrogenation temperature of 500 °C and a dehydrogenation time of 5 h. The dehydrogenated powder was further refined by a jet milling (JM) process, resulting in an average powder size of 3.15 µm. Then, the RP is subjected to orientation molding and sintering to obtain the first-generation regenerated magnets (RM-I). A batch of perfect jet mill powder was selected as the repair agent in the sintered Nd-Fe-B production line of the factory and named nascent Nd-Fe-B powder (NP). Its nominal composition is shown in Table 1, characterized by a slightly higher rare earth content (RE ≥ 31 wt%) and a particle size close to RP at 3.35 µm. The NP was mixed with RP at 1 wt%, 2 wt%, and 3 wt% substitutions, respectively, and then mixed for 3 h. The mixed powder was orienting compression under a 2 T magnetic field, and finally, the second-generation regenerated magnets (RM-II) were prepared by vacuum sintering. The process is shown in Figure 1b.

### 2.2. Analytical Techniques

The preparation process of the magnets relied on the Nd-Fe-B production line without other experimental operations. The elemental composition of the RP was determined using an inductively coupled plasma spectrometer (ICP, Pride-100, Beijing Huake Tiancheng technology Co., Ltd., Beijing, China). The magnetic properties were evaluated using the magnetic measurement system (NIM-500C, National institute of metrology, Beijing, China) with a testing temperature of 25 °C. The magnet densities were measured by using the Archimedes means. The weight of the magnet in air, m1, was tested first, and then the weight of the magnet in pure water, m2, was tested, and the density of the magnet was obtained by dividing m1 by m2. To ensure the accuracy of the data, the surface of the magnets was smoothed before testing, and each sample was also tested five times before taking the average value. The microstructure of the regenerated magnets was observed using a scanning electron microscope (SEM, MIRA3-LMH, TSECAN, Shanghai, China), and the elemental distribution at grain boundaries and within the main phase was analyzed using the accompanying EDS module. The proportion of Nd-rich phases within multiple randomly selected areas at low magnification of the magnet was separately counted using the open-source software package ImageJ (Version 1.54h, https://imagej.net/ij/ (accessed on 5 July 2024)). The thermal behavior of the samples was investigated using a differential scanning calorimeter (DSC, STA449F3, NETZSCH, Shanghai, China) under a protective atmosphere of Ar gas flowing at 100 mL/min, with a heating rate of 10 °C/min to obtain the TG-DSC curves. The phase constitution was examined by X-ray diffraction (XRD, Empyrean, Malvern Panalytical, Shanghai, China) using Cu–Kα radiation. The electrochemical measurements were conducted using an electrochemical workstation (PARSTART 4000, AMETEK Scientific Instruments, Shanghai, China), employing a traditional three-electrode system composed of an Nd-Fe-B working electrode, a saturated calomel reference electrode, and a platinum counter electrode. The experiments were carried out in a 3.5 wt% NaCl solution at a temperature of 25 ± 0.2 °C. After stabilizing the open-circuit potential for 30 min, polarization curves were recorded at a scan rate of 2 mV/s.

## 3. Results and Discussion

### 3.1. Subsection

Due to the wide range of sources and complex composition of recycled waste magnets, understanding the elemental distribution of recycled magnets is crucial. Table 1 presents the composition of the RP prepared through hydrogen decrepitation and jet milling processes. From the table, it can be observed that apart from the common Pr and Nd elements, the RP also contains 6.3 wt% of Ce element. The total rare earth content is 28.4 wt%, which is significantly lower than the commonly mentioned 30 wt% in the composition design principles for sintered Nd-Fe-B magnets. Additionally, the content of the B element in the composition is also lower than the normal design level (≤1.03 wt%) [27]. This is attributed to the repetitive crushing process involved in the recycling of waste magnets, as most Nd-Fe-B materials are prone to intergranular fracture, leading to loss at grain boundaries during this process. Furthermore, due to the higher rare earth content at grain boundaries, corrosion is more likely to occur during the use of magnets. These factors contribute to the lower rare earth proportion in the final RP [18,20]. However, for sintered Nd-Fe-B magnets, optimizing the structure and distribution of the grain boundary phase is crucial. On the one hand, it can enhance liquid-phase sintering and improve magnet density, and the higher rare earth content in the grain boundary phase promotes the formation of more primary phases. On the other hand, the grain boundary phase can form a continuous thin-layer structure to isolate primary phase grains, achieving a demagnetizing effect [3,28]. Therefore, we have chosen the NP with a relatively higher rare earth content to dope, aiming to restore the grain boundary structure of the regenerated magnets through a greater proportion of the Nd-rich phase, the nominal composition of which is also provided in Table 1.

### 3.2. The Effect of NP Substitution on the Regenerated Magnets

**Figure 2:**
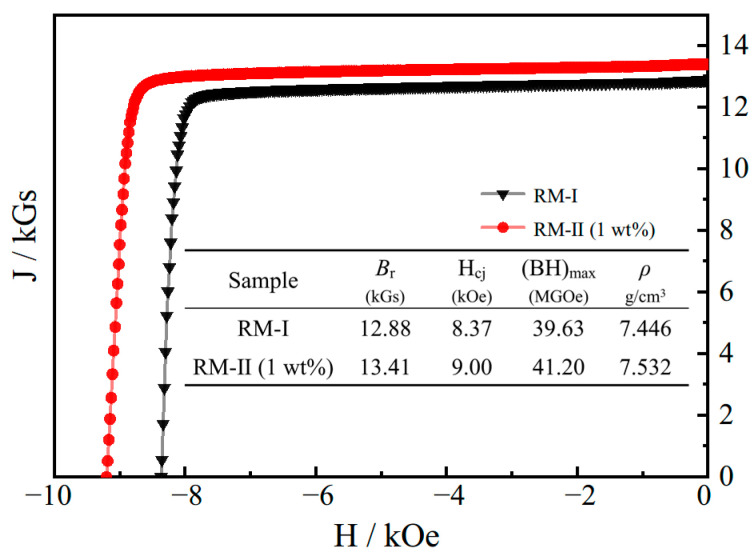
The demagnetization curves and corresponding magnetic properties of the RM-I and RM-II magnets.

Figure 2 shows the demagnetization curves of the RM-I magnet, directly sintered at 1075 °C using RP, and the RM-II magnet, which was sintered after mixing with 1 wt% of NP. The embedded table in Figure 2 lists various magnetic performance indicators. The remanence (*B*_r_) of the RM-I magnet is 12.88 kGs, with a slightly lower coercivity (*H*_cj_) of only 8.73 kOe. In contrast, the RM-II magnet exhibits significant improvements in remanence, coercivity, and magnetic energy product, reaching 13.41 kGs, 9.0 kOe, and 41.20 MGOe, respectively. This indicates that the addition of NP has a positive effect on the preparation of regenerated magnets.

**Figure 3:**
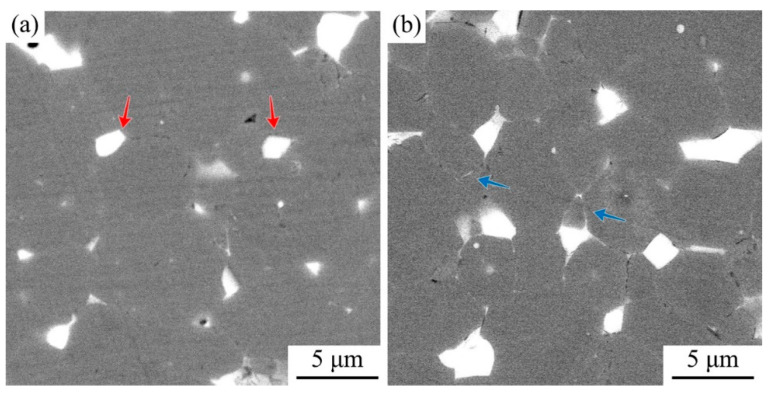
The microstructure of the RM-I magnet (**a**) and the RM-II magnet (**b**) sintered at 1075 °C.

Figure 3 shows the microstructure of the first and second generations of the regenerated magnets. In Figure 3a, it can be observed that the Nd-rich phase proportion in the RM-I magnet is relatively low, and it is clustered and filled in the triangular grain boundaries, as indicated by the area pointed out by the red arrows. Additionally, thin grain boundaries are scarcely visible. The scarcity of this structure can lead to direct contact between the main grain boundaries, resulting in strong exchange coupling and subsequently reducing coercivity [29]. Furthermore, the lower proportion of the Nd-rich phase can enhance the effect of solid-phase sintering, leading to grain growth and inadequate density. Therefore, the densities of the two samples were measured using the drainage method, and the results are presented in Figure 2. As expected, the density of the RM-I magnet is 7.446 g/cm^3^, which is lower than that of the RM-II magnet at 7.532 g/cm^3^. The higher density is one of the reasons for the increased residual magnetization in the RM-II magnet. Another reason is that the saturation magnetization of Nd_2_Fe_14_B is greater than that of Ce_2_Fe_14_B, leading to the increased proportion of Nd_2_Fe_14_B when NP is added [30]. Upon examining the RM-II magnet, a significant increase in the proportion of the Nd-rich phase is clearly evident. To validate this result, we employed the open-source software package ImageJ to randomly analyze the proportions of the Nd-rich phase in the two magnets. The study results indicate that after the addition of NP, the proportion of the Nd-rich phase in the RM-II magnet increased from the original 5.389% to 6.086%. The introduction of a more Nd-rich phase resulted in the formation of a greater number of fine-grain boundary structures in the RM-II magnet. As indicated by the area pointed out by the blue arrows in Figure 3b, these fine-grain boundary structures can effectively isolate the main phase grains, thereby weakening the exchange coupling and consequently enhancing coercivity.

### 3.3. Effect of Sintering Temperature on the Regenerated Magnets

**Figure 4:**
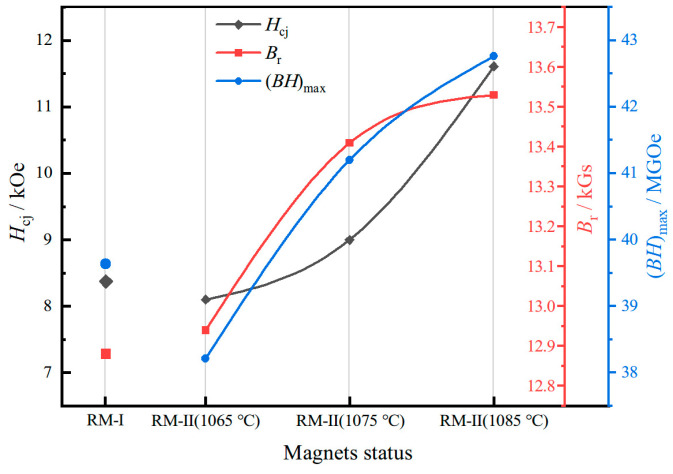
The variation curve of magnetic properties of regenerated magnets at different sintering temperatures.

The microstructure of the magnet, particularly the Nd-rich phase, is closely associated with the sintering process [31,32]. To further optimize the magnetic properties of the second-generation regenerated magnet, we investigated the sintering temperature of the RM-II (1 wt%) magnet. Specifically, we varied the sintering temperature to 1065 °C and 1085 °C, each maintained for 6 h, to prepare two new process variants of the RM-II magnet. Figure 4 presents the variations in the magnetic properties of each magnet. It is evident from the figure that when the sintering temperature is reduced to 1065 °C, both coercivity, residual magnetization, and magnetic energy product decrease to 8.10 kOe, 12.94 kGs, and 38.21 MGOe, respectively. Conversely, when the sintering temperature is increased to 1085 °C, the coercivity increases to 11.61 kOe, residual magnetization increases to 13.53 kGs, and the maximum magnetic energy product increases to 42.76 MGOe. It is noteworthy that for the RM-II magnet when the sintering temperature is reduced to 1065 °C, its coercivity and maximum magnetic energy product are even lower than those of the RM-I magnet, which is disadvantageous for our optimization goal.

**Figure 5:**
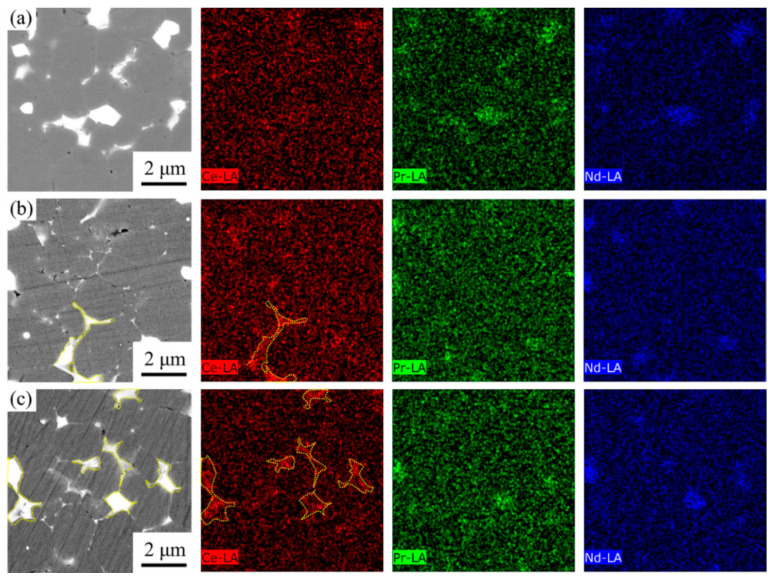
The SEM images and corresponding ESD surface scans of regenerated magnets at different sintering temperatures: (**a**) RM-II (1065 °C), (**b**) RM-II (1075 °C), (**c**) RM-II (1085 °C).

The SEM images in Figure 5 provide crucial insights into the microstructural changes of the three different types of magnets under inspection. The magnet in Figure 5a, sintered at a temperature of 1065 °C, predominantly exhibits a congregation of substantial rare earth phases situated in the triple junctions within the magnet. This distribution is comparable to the Nd-rich phase distribution in the RM-I magnet. Unfortunately, such structures prove to be deficient when it comes to isolating the exchange coupling among the main phase grains, resulting in a decline in coercivity. Conversely, a magnet sintered at an elevated temperature of 1085 °C demonstrates the significant presence of sleek, continuous fine-grain boundaries, as depicted in Figure 5c. These boundaries play an indispensable role in magnetization de-coupling, thus leading to an augmentation in the overall coercivity of the magnet. Upon observing the corresponding EDS surface scans on the right, it can be noted that, in the RM-I magnet sintered at 1065 °C, the Ce element is dispersed within the magnet, while Pr and Nd elements have slightly concentrated at grain boundaries. As the sintering temperature elevates, the Ce element gradually enriches at the grain boundaries while the Pr element diffuses throughout the magnet, thus enhancing Pr concentration in the main phase. In other words, when the sintering process occurs at elevated temperatures, there is a diffusion of Pr into the main phase, which in turn replaces the Ce. These replaced Ce elements then accumulate at the grain boundaries. A comparison between Figure 5b,c reveals that this diffusion effect is more pronounced with increasing sintering temperature. Given that the anisotropy and saturation magnetization properties of Pr_2_Fe_14_B exceed those of Ce_2_Fe_14_B, there is an enhancement in the remanence and coercivity of the second-generation regenerated magnets with the elevation in sintering temperature.

**Figure 6:**
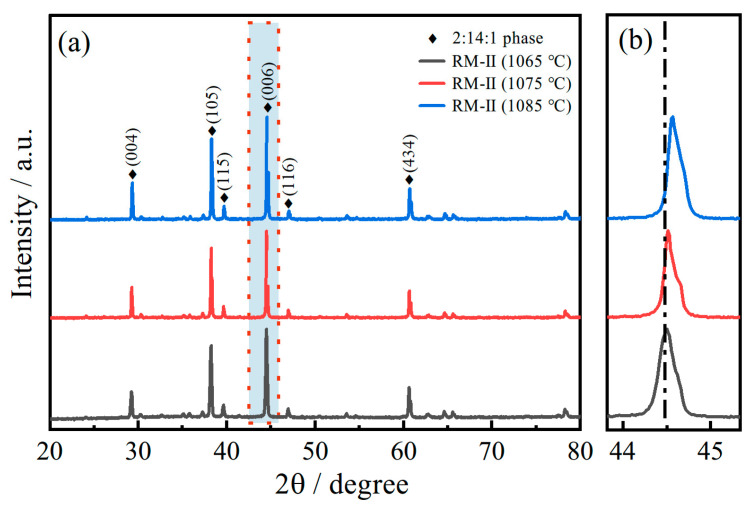
The XRD pattern of the examined RM-II magnets at different sintering temperatures (**a**) and a zoomed-in view of the red area (**b**).

The XRD pattern of the examined samples of RM-II magnets displayed in Figure 6 predominantly contains peaks corresponding to the hard magnetic RE_2_Fe_14_B (i.e., 2:14:1) phase (space group P42/mnm) with the tetragonal structure, which can be indexed to the pdf #36–1296. The characteristic peak height of the three samples remains consistent, with no other significant peaks identified. Further examination of the main (006) peak reveals a shift towards larger angles as the sintering temperature increases. According to Bragg’s law (nλ = 2dsinθ), an increase in diffraction peak angle denotes a decrease in interplanar spacing [30,33]. This occurs as the larger atomic radius of Ce (2.7 Å) is replaced by the smaller Pr (2.67 Å), causing a contraction in the lattice volume and consequentially reducing the interplanar distance. This aligns with the diffusion phenomena of Pr elements revealed in the previous Figure 5.

**Figure 7:**
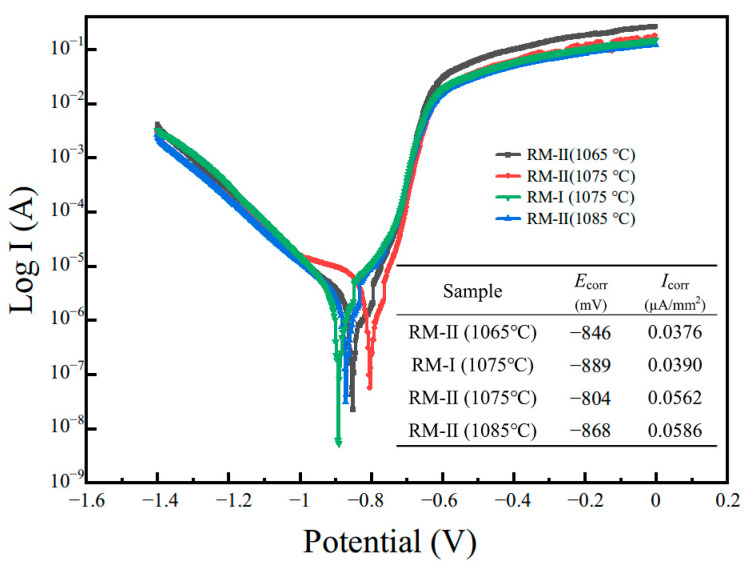
The Tafel curves of the regenerated magnets at different sintering temperatures.

Figure 7 displays the Tafel curves of the regenerated magnets, and the corresponding self-corrosion potentials and corrosion current densities are also listed in the table. A comparison of the RM-I and RM-II magnets sintered at 1075 °C reveals that the magnets with NP are more vulnerable to corrosion, and the corrosion potential ascends from −889 mV to −804 mV. Concurrently, the corrosion current density also climbs from 0.039 µA/mm^2^ to 0.562 µA/mm^2^, and the corrosion rate is greatly enhanced. In general, the corrosion behavior of an Nd-Fe-B sintered magnet is closely associated with the microstructure of the magnet’s grain boundaries. The above phenomenon is due to the addition of NP, which raises the proportion of rare earth elements, improves the continuity of the grain boundaries, and thus amplifies the intergranular corrosion of the material. When the sintering temperature is lowered, the corrosion potential further drops from −804 mV to −846 mV, and the corrosion tendency is diminished. At the same time, the corrosion current density also reduces to 0.0376 µA/mm^2^. This is caused by the degradation of the grain boundary continuity. On the other hand, when the sintering temperature is increased to 1085 °C, the continuity of the grain boundaries is markedly improved, the corrosion channel of the material is smoother, and the corrosion current density surges.

### 3.4. The Effect of NP Substitution Amount on the Regenerated Magnets

**Figure 8:**
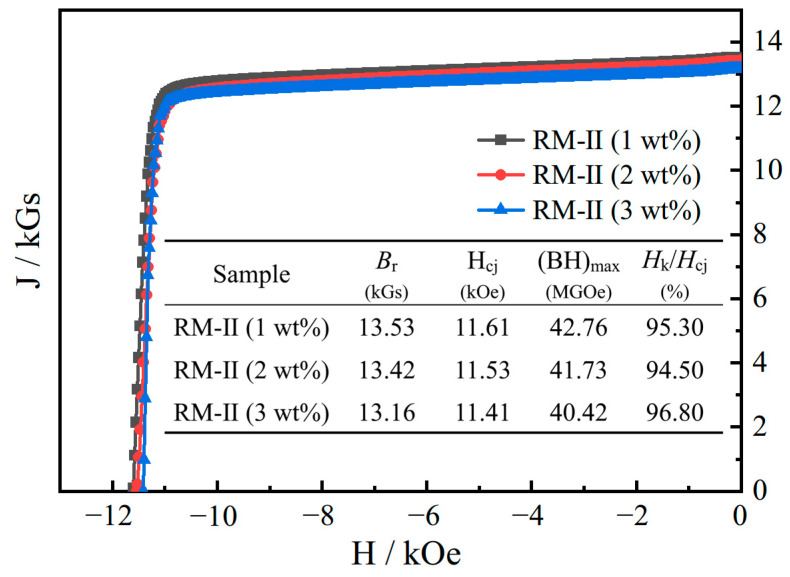
The demagnetization curves and corresponding performance indicators of the second-generation regenerated magnets with different additions of NP sintered at 1085 °C: RM-II (1 wt%), RM-II (2 wt%), and RM-II (3 wt%).

In pursuit of augmenting the performance of regenerated magnets, the strategy undertaken involved supplementing additional NP aiming to upgrade the grain boundary structures. As demonstrated in Figure 8, the demagnetization curves for the RM-II magnets, fabricated by the incremental addition of NP (1 wt% and 2 wt%) predicated on the sintering process at 1085 °C, are nearly identical. As can be gleaned from the table within the figure, a subtle decrement was observed in the remanence, coercivity, and maximum energy product, with the escalating quantity of NP added (3 wt%). The variance in remanence quantified to 0.37 kGs and coercivity to 0.2 kOe, representing minuscule magnitudes for the sintered Nd-Fe-B materials. Thus, it can be ascertained that despite the increment in NP quantity, the magnetic properties remained uninfluenced.

**Figure 9:**
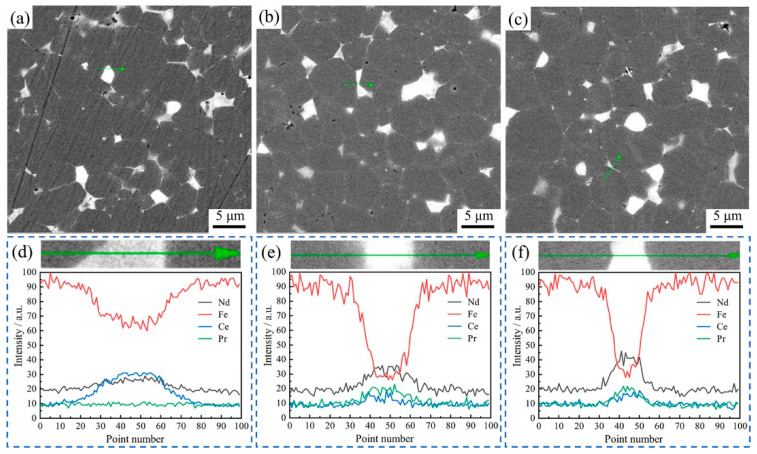
The SEM images of the second-generation regenerated magnets with different additions of NP sintered at 1085 °C: (**a**) RM-II (1 wt%), (**b**) RM-II (2 wt%), and (**c**) RM-II (3 wt%), and the analysis of EDS elements in the area indicated by the arrow: (**d**) RM-II (1 wt%), (**e**) RM-II (2 wt%), and (**f**) RM-II (3 wt%).

To discern the underlying cause of the observed alterations in magnetic properties, this study conducted a comparative analysis of the microstructural disparities and variations in grain boundary phase compositions of RM-II magnets under different conditions. These findings are depicted in Figure 9. As evidenced by the SEM images in Figure 9a–c, there are no substantial microstructural variations across the three magnet types. However, a meticulous examination reveals that as the proportion of NP increases, the grain boundaries become progressively indistinct, which undoubtedly impacts the coercivity of the magnets. An EDS line scans were executed on the grain boundary regions of the three magnet samples, and the results are illustrated in Figure 9d–f, correspondingly representing RM-II (1 wt%), RM-II (2 wt%), and RM-II (3 wt%) magnets. These results show that RM-II (1 wt%) magnets had a notably elevated concentration of Ce element at the grain boundary. Conversely, the concentrations of Pr and Nd elements were comparatively low. This finding corroborates the reactions depicted in Figure 5, which occurred when the Pr element infiltrated the primary phase, displacing the Ce element and facilitating its movement toward the grain boundary. In contrast, an examination of RM-II (2 wt%) and RM-II (3 wt%) magnets revealed no discernible accumulation of Ce element within their grain boundaries. Instead, Nd and Pr elements demonstrated higher concentrations than Ce, suggesting that an increase in the addition quantities of RM-II (2 wt% and 3 wt%) did not result in the displacement of Ce as observed in the RM-II (1 wt%) magnets.

**Figure 10:**
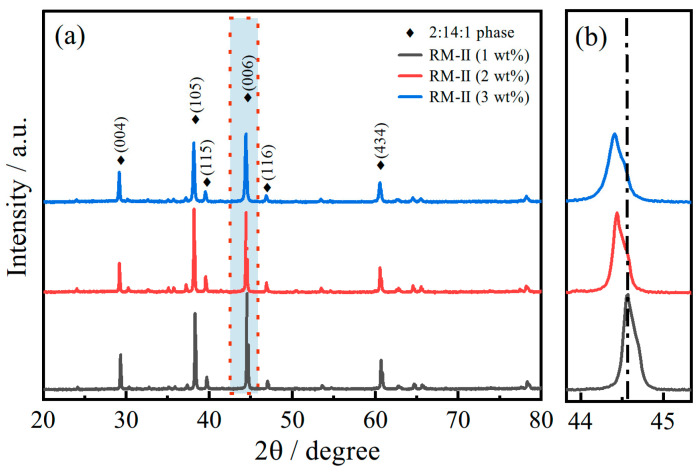
XRD spectra of RM-II magnets with different amounts of NP added (**a**) and a zoomed-in view of the red area (**b**).

The XRD analysis was conducted on three samples, and the results are shown in Figure 10. Despite the addition of more NP, the major diffraction peaks remained unchanged. However, it is noteworthy that with an increase in the amount of added powder, there was an overall shift towards smaller diffraction angles. Based on the previous testing shown in Figure 6 for the RM-II (1 wt%) magnet, it was established that the diffraction peaks shifted towards larger angles due to the substitution of Ce by Pr. Conversely, in comparison with the substituted magnet, the diffraction peaks of the unsubstituted magnet should shift towards smaller angles. From the results depicted in Figure 10, it is evident that with an increasing amount of added powder, the overall diffraction peaks shifted towards smaller angles, indicating that the substitution of Ce did not occur with the addition of more powder. This conclusion aligns with the findings of Figure 9. Consequently, it can be inferred that when preparing recycled magnets using a sintering process at 1085 °C, adding more NP does not lead to an optimized grain boundary structure for enhanced coercivity. Furthermore, it does not promote the substitution of Ce to enhance remanence and coercivity.

**Figure 11:**
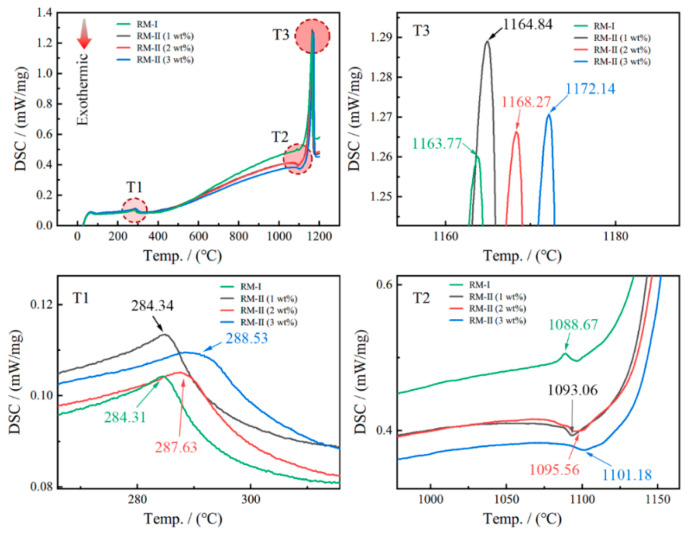
The DSC curves of the RM-I magnet and the RM-II magnets with different amounts of NP added.

Incorporating a 1 wt% addition of NP at varying sintering temperatures results in distinct performance outcomes. Surprisingly, maintaining a constant sintering temperature while adjusting the powder amount leads to an unexpected decrease in magnetic performance, contrary to the anticipated enhancement. This perplexing phenomenon can be attributed to the combined influence of differential grain boundary repair and elemental interdiffusion effects. Therefore, a comprehensive investigation of the metallurgical behavior is essential to elucidate the underlying causes of this observed behavior. Figure 11 illustrates the DSC curves ranging from 20 °C to 1200 °C for the RM-I and RM-II magnets with varying amounts of NP. Notably, three distinct temperature regions, denoted as T1, T2, and T3, exhibit substantial disparities, warranting further analysis. Within the T1 temperature range, all samples exhibit an endothermic peak corresponding to the Curie temperature (Tc) of the respective samples. Although the Tc values of RM-II and RM-I magnets show minimal disparity, an incremental rise in Tc is observed with increasing amounts of NP in the RM-II magnets. This phenomenon can be attributed to the increased proportion of NP in the magnet, that is, the increased proportion of Nd_2_Fe_14_B, which has a higher Tc. Moving to the T2 temperature range, intriguing observations emerge. The RM-I magnet exhibits an endothermic peak at 1088.67 °C. However, upon the addition of NP, the endothermic reaction transforms into an exothermic reaction. Furthermore, the magnitude of the exothermic peak proportionally increases with the quantity of NP added. This could be attributed to the reaction between the decomposed oxygen in the regenerated powder and the highly reactive Nd-rich Nd in the nascent powder, resulting in the formation of a more stable oxide. The poor mobility of the oxide hinders grain boundary repair by NP. Moreover, within the T3 temperature region, the RM-I magnet displays a relatively low melting point of 1163.77 °C, which gradually rises with an increasing amount of NP. This suggests that higher sintering temperatures are required for the NP. Therefore, at a sintering temperature fixed at 1080 °C, despite the increase in NP, the repair of grain boundaries was not better achieved due to the temperature mismatch. Consequently, as NP is incrementally introduced, further refinements in the sintering process become imperative to facilitate the intrinsic repair of the grain boundary structure by the NP crystals. In conclusion, this investigation highlights the intricate interplay between sintering temperature, NP content, and resultant magnetic performance. The observed deviations in properties necessitate a comprehensive understanding of the metallurgical behavior, enabling the development of optimized sintering methodologies and the promotion of effective grain boundary repair within regenerated magnets.

## 4. Conclusions

This study utilized existing Nd-Fe-B production equipment to transform various waste magnets into recycled Nd-Fe-B powder. Additionally, a nascent Nd-Fe-B powder with slightly higher rare earth content was selected from the production line as a restorative agent for the regenerated magnets. By incorporating the nascent Nd-Fe-B powder into the recycled Nd-Fe-B powder, the regenerated magnets were produced.

The results show that the nascent Nd-Fe-B powder has a restorative effect on the grain boundaries of the regenerated magnets. And this effect is better when the sintering temperature is optimized. For instance, at a sintering temperature of 1085 °C and with a 1 wt% addition of nascent Nd-Fe-B powder, the proportion of the Nd-rich phase in the magnet increased, and a continuous and clear fine grain boundary structure was observed. Additionally, the Pr element in the nascent magnetic powder was able to replace the Ce in the main phase, thereby improving the intrinsic performance. In conclusion, after the repair of the nascent magnetic powder and the optimization of the sintering temperature, the coercivity, the remanence, and the magnetic energy product of the magnets have been comprehensively improved. However, when the addition of nascent Nd-Fe-B powder continued to be increased, an opposite situation to what was expected occurred and the magnetic properties were not optimized. It was detected that the grain boundary structure was not repaired with the addition of more nascent Nd-Fe-B powder, and the substitution of Ce element did not occur. This is due to the fact that the nascent magnetic powders require higher sintering temperatures, so the repair of grain boundaries is not better achieved. Therefore, in future work to improve the performance of regenerated magnets, the sintering temperature needs to be continuously adjusted to accommodate different amounts of fresh Nd-Fe-B powder to promote the restoration effect of fresh Nd-Fe-B powder.

## Figures and Tables

**Table 1:** The normalized distribution of each element in the RP and NP.

Types	Pr	Nd	Gd	Ce	B	Co	Cu	Al	Ga	Zr	Ti	Fe
RP	4.2	17.9	/	6.3	0.96	0.5	0.3	0.3	0.1	0.15	0.1	bal
NP	7.325	21.985	2	/	0.94	0.8	0.3	0.35	0.2	0.1	0.1	bal

## Data Availability

The original contributions presented in the study are included in the article, further inquiries can be directed to the corresponding authors.
